# Toxicokinetics of Kava

**DOI:** 10.1155/2011/326724

**Published:** 2011-03-21

**Authors:** Anthony Rowe, Lillian Yuan Zhang, Iqbal Ramzan

**Affiliations:** Faculty of Pharmacy, University of Sydney, Bank Building (A15), Sydney, NSW 2006, Australia

## Abstract

Kava is traditionally consumed by South Pacific islanders as a drink and became popular in Western society as a supplement for anxiety and insomnia. Kava extracts are generally well tolerated, but reports of hepatotoxicity necessitated an international reappraisal of its safety. Hepatotoxicity can occur as an acute, severe form or a chronic, mild form. Inflammation appears to be involved in both forms and may result from activation of liver macrophages (Kupffer cells), either directly or via kava metabolites. Pharmacogenomics may influence the severity of this inflammatory response.

## 1. Introduction

Kava (*Piper methysticum* Frost F.) is a perennial plant which has been used for centuries by South Pacific communities for medicinal, social, and cultural purposes. Traditionally, the rhizome of the plant is macerated with water or coconut milk to produce a beverage with relaxant and psychoactive properties [1]. In the 20th century, it became popular in the Western world as a herbal supplement for anxiety and insomnia.

## 2. Chemistry and Toxicology of Kava

More than 40 compounds have been isolated from kava, with the active components present in the lipid-soluble resin containing three chemical classes (i) arylethylene-*α*-pyrones, (ii) chalcones and other flavones, and (iii) conjugated diene ketones. It is the substituted 4-methoxy-5, 6-dihydro-*α*-pyrones or kavapyrones commonly known as kavalactones that possess the highest anxiolytic effects [2].

**Figure 1:**
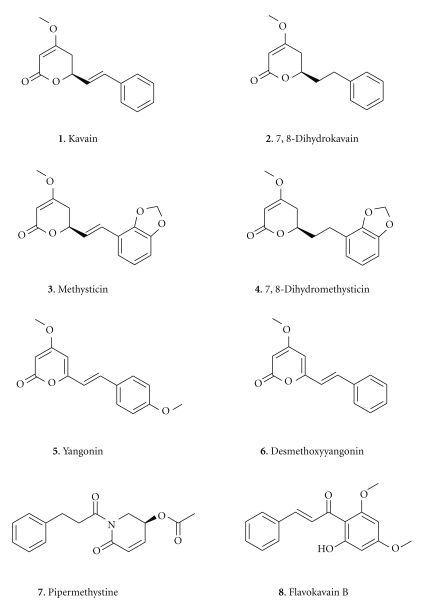
Structures of the six main kavalactones **(1–6)**, Pipermethysticine **(7)**, and Flavokavain B **(8)**. The chemical formulae are kavain (C_14_H_14_O_3_), 7,8-dihydrokavain (C_14_H_16_O_3_), methysticin (C_15_H_14_O_5_), 7,8-dihydromethysticin (C_15_H_16_O_5_), yangonin (C_15_H_14_O_4_), desmethoxyyangonin (= 5,6-dehydrokavain = C_14_H_12_O_3_), pipermethystine (C_16_H_17_NO_4_), and flavokavain B (C_17_H_16_O_4_).

Total kavalactone accounts for 3%–20% dry weight, with the highest concentration in the lateral roots, decreasing gradually towards the aerial plant structures. To date, eighteen kavalactones have been identified from the root, six of which account for approximately 95% of the organic extract namely, kavain, dihydrokavain, methysticin, dihydromethysticin, yangonin, and desmethoxyyangonin (Figure 1). When these kavalactones are eluted from a sample of kava by HPLC and sorted by decreasing order of quantity, the chemical signature obtained distinguishes individual strains (cultivars). Such chemotyping has identified over 200 variant strains of kava, but the chemical signature can vary between roots, rhizomes, and basal stems [3]. Root and leaf extracts from four Hawaiian cultivars (Mahakea, PNG, Purple Moi, and Nene) found that dihydrokavain and dihydromethysticin comprised more than 70% of the total kavalactones in leaves, whereas each of the six major kavalactones represented around 10% to 20% of the total kavalactones in root extracts [4].

Whilst kava extracts are generally well tolerated, toxic doses were determined *in vivo* using animal studies and *in vitro*. The LD_50_ (mg/kg) for kavain, methysticin, dihydrokavain, and dihydromethysticin in mice ranges from 41–69 (intravenous), 325–530 (intraperitoneal), and 920–1130 (oral) [2]. F344 rats treated with 2 g/kg/day kava extracts in corn oil by oral gavage five days a week for fourteen weeks exhibited elevations in *γ*-glutamyl transferase (GGT), serum cholesterol, protein, and albumin levels, along with hypoglycaemia, within days of treatment [5]. Cytotoxicity associated with kavalactones was demonstrated for human hepatocytes *in vitro* (EC_50_ values approximately 50 *μ*M) [6] and neurones *in vivo* at ≥300 *μ*M [7], and apoptosis is the mechanism of cell death [8]. Since the highest serum kavain concentration recorded in a human is 17.4 *μ*M [9], these data suggest that for most individuals, kavalactones have a wide therapeutic index.

Pipermethysticine (PM) is a toxic alkaloid present in kava leaves and stem peelings, which can contaminate kava products during high production and/or poor quality control [10]. HepG2 human hepatoma cells exposed to 100 *μ*M PM for 24 hours showed 90% loss of cell viability, with 65% loss of viability with 50 *μ*M PM due to disruption of mitochondrial function and consequent apoptosis [11]. Fischer-334 rats given 10 mg/kg PM daily by oral gavage for two weeks demonstrated adaptive changes to oxidative stress such as a significant increase in hepatic glutathione and cytosolic superoxide dismutase (Cu/ZnSOD) [12]. Thus, PM is a potential cause of kava hepatotoxicity although it was not detected in any samples in a recent study using products available on the German market [13].

Flavokavain B is a cytotoxic component of kava root and is present in aqueous and organic extracts [14]. The highest yields are in chloroform extracts, followed by acetone then hexane fractions [15]. Hepatocellular toxicity results from mitogen-activated protein kinase (MAPK) signalling leading to oxidative stress and apoptosis [16]. Due to its location in the plant root, flavokavain B is likely to be present in both traditional and pharmaceutical preparations of kava. 

The chemical content of kava products and hence potential for adverse effects vary according to plant age, part used, cultivar, geographical location, and growth conditions. In the Kava Act 2002 [17] the government of Vanuatu classified the different cultivars of kava as noble (traditional social beverage with long history of safe use), medicinal (used for specific therapeutic effects), two days (can cause strong side effects such as nausea due to high levels of the kavalactone dihydromethysticin and banned for export), and wichmannii (which also induce strong side effects and are banned from export) [18]. 

Emphasis on the links between method of preparation and toxicity has declined recently. This is due to reports identifying adverse effects in patients using either ethanolic/acetonic kava extracts or traditional aqueous extracts, suggesting that toxicity is more dependent on the kava plant itself than the extraction solvent(s) used [19].

## 3. Adverse Effects of Kava

Meta-analyses of placebo-controlled studies showed a significant reduction in anxiety for patients receiving kava extract compared with patients receiving placebo [20, 21]. The most common treatment regime used was 300 mg/day for four weeks. A study using a similar dose (280 mg/day for four weeks) found no difference between kava extract and placebo in terms of occurrence of adverse events, withdrawal symptoms, effect on heart rate, blood pressure, laboratory assessments, and sexual function, which supports the safety of this protocol [22].

However, two post-marketing trials in Germany (*n* > 3000) showed a small dose-dependent increase in adverse effects caused by kava extracts [23]. Risk factors for adverse reactions include chronic, heavy use and concurrent use with other drugs, herbs, and dietary supplements [24, 25].

Side effects of kava consumption include skin reactions and central nervous system effects. Kava dermopathy has been well documented among Pacific Islanders [26] and is a reversible condition characterised by dry scaly yellow skin covering the palms of the hands, soles of the feet, and back [27]. It is speculated to have a link to cholesterol metabolism and hepatotoxicity due to the presence of jaundice [28]. Short-term kava use can produce extrapyramidal side effects resulting in oral dyskinesia and serious exacerbations of parkinsonism-like symptoms, while heavy use may predispose individuals to seizures [27]. However, a double-blind placebo controlled trial showed kava has no effect on motor vehicle performance. In another study, no statistically significant difference between kava and placebo was present in participants performing tracking tasks [27].

The most serious adverse reaction associated with kava is hepatotoxicity. Reports of kava hepatotoxicity first emerged in Germany in 1998, and by the end of 2005, the World Health Organisation had received 91 reports of 189 adverse reactions relating to kava-only products. Fiftly-five of those reactions involved liver and biliary system disorders, including three cases of hepatic failure and two cases of hepatic comas [29]. Reported daily doses ranged from 45–1200 mg kavalactones taken for one week to twelve months [25].

## 4. Metabolism of Kavalactones

The main metabolic pathways for kavalactones in humans and rats are hydroxylation of the C-12 in the aromatic ring, breaking and hydroxylation of the lactone ring with subsequent dehydration, reduction of the 7,8-double bond, and demethylation of the 4-methoxyl group [30–32]. Products of kavain metabolism found in human serum and urine include *p*-hydroxykavain, *p*-hydroxy-5,6-dehydrokavain, *p*-hydroxy-7,8-dihydrokavain, 5,6-dehydrokavain, 6-phenyl-5-hexen-2,4-dione [33], and 6-phenyl-3-hexen-2-one [34]. Human metabolites identified for other kavalactones include 11,12-dihydroxykavain-*o-*quinone for methysticin, 11,12-dihydroxy-7,8-dihydrokavain-*o-*quinone for 7,8-dihydromethysticin and 12-desmethylyangonin either from demethylation of the 12-methoxyl group of yangonin or hydroxylation at C-12 of desmethoxyyangonin [30, 35]. In rats, approximately 50% to 75% of administered kavalactones are excreted in the urine, mostly as glucuronide and sulphate conjugates. Approximately 15% is excreted in the bile [30, 32, 36]. Reactive kava metabolites may potentially alkylate DNA or disrupt enzymatic and metabolic activity, inducing hepatotoxicity.

## 5. Mechanism of Kava Hepatotoxicity

Despite the evidence for liver damage and inflammation in animals and humans treated with kava, clinical cases of hepatotoxicity amongst indigenous users caused by traditional aqueous root extracts are limited to two cases in New Caledonia [37]. The dose of kavalactones in one patient was 18 grams per week for four to five weeks and was unknown in the other. Clinical surveillance in the Northern Territory, Australia over 20 years has not documented any cases of fulminant hepatic failure attributable to kava, despite doses estimated to be 10–50 times the recommended therapeutic doses. 

Based on these observations, most hepatic effects of kava appear to be reversible or can be compensated for. Hence, pharmacogenomic effects may be involved in the most severe cases of toxicity. For example, the hydroxylation of the aromatic ring and demethylation of kavalactones is a function of CYP2D6 enzymes [30]. Four human phenotypes for CYP2D6 activity (ultrarapid, efficient, intermediate, and poor) are defined according to debrisoquine metabolism. The two Europeans with kava-related hepatotoxicity were phenotyped as poor metabolisers according to CYP2D6 activity [38]. It is known that 12%–21% of Caucasians are poor metabolisers compared to less than 1% for Asians/Pacific Islanders, which may contribute to the lower incidence of kava hepatotoxicity in the Pacific [39]. Caucasians also have a higher frequency of ultrarapid metabolisers compared to Asians/Pacific Islanders (1% to 5% versus 0% to 2%). These individuals could experience adverse reactions following a burst of reactive kava metabolites. 

Acute kava hepatotoxicity involves inflammation. Histopathology results from a 50-year-old man [40] and a 33-year-old woman [38] displaying hepatic symptoms following use of kava supplements showed extensive, severe hepatocellular necrosis and infiltration with lymphocytes, eosinophils, and activated macrophages. Experimental studies with kavain-perfused rat livers examined via electron microscopy displayed a disruption of hepatic vasculature with narrowing of blood vessels, constriction of sinusoidal blood vessels, and retraction of the endothelium compared to controls [41]. Liver macrophages (Kupffer cells) within the sinusoids of the kavain-perfused liver also appeared swollen with large cytoplasmic vacuoles and phagocytosed material.

Subclinical liver abnormalities occurred in clinical studies with chronic, indigenous, kava users. A study with Australian aborigines in the Northern Territory revealed elevations in *γ*-glutamyl transferase (GGT) and alkaline phosphatase (ALP) in 61% and 50%, respectively, of participants who reported using kava at least once in the month prior to the measurement, compared to less recent users and nonusers [42]. These enzymes return to normal after one to two months of abstinence [43]. There were no differences between the groups in ALT, bilirubin, albumin, or total protein. Unlike the German clinical trials for anxiety, kava consumption was not regulated in these studies, and the median duration of kava use was twelve years, with a range from one to eighteen years.

Similar results were observed in a predominantly Tongan population in Hawaii, which compared liver function tests between 31 healthy adult kava beverage drinkers and 31 healthy adult nonkava beverage drinkers [44]. GGT was significantly elevated in 65% of the kava drinkers versus 26% in the controls, and ALP was significantly elevated in 23% of kava drinkers versus 3% in the controls. There was no significant difference in ALT, AST, bilirubin, albumin, or total protein. Increases in GGT and ALP without ALT or AST elevation are suggestive of cholestasis rather than hepatocellular damage.

Cholestasis can be due to either defective bile formation in hepatocytes or disruption to bile secretion and flow within bile ducts [45]. Mechanisms of noninflammatory cholestasis include inhibition of cellular proteins and transporters. Direct or indirect activation of Kupffer cells and their subsequent release of pro-inflammatory cytokines, growth factors, and reactive oxygen species is a cause of inflammatory cholestasis, which may involve hepatocytes or bile ducts [46]. In the absence of hyperbilirubinaemia, elevations in GGT and ALP are more likely to be due to bile duct inflammation. Each of these mechanisms could be precipitated by kava extracts and are possible explanations for the cholestasis observed in chronic, indigenous, kava users.

Hence, Kupffer cells could be involved in both acute and chronic kava hepatotoxicity. These cells are also implicated in the pathogenesis of many other liver conditions, including fibrosis, viral hepatitis, steatohepatitis, alcoholic liver disease, and activation or rejection of the liver during transplantation [47, 48]. In animal studies, depletion of the Kupffer cell population is hepatoprotective during ischemia repurfusion injury [49], sepsis [50, 51], radiotherapy [52], and diet-induced steatosis and insulin resistance [53]. Kupffer cell depletion experiments could be a valuable tool for determining their role in kava hepatotoxicity.

## 6. Additional Studies

Furthur studies could investigate the extent of kavalactone metabolism in humans and the proportions of urinary versus biliary excretion of kavalactones and their metabolites at different time points after ingestion. Such studies would further define normal kavalactone metabolism, suggest target cytochrome P450 enzymes for pharmacokinetic herb-drug interactions with kavalactones, and aid the identification of toxic metabolite(s) and their contribution to kava hepatotoxicity. Similar animal studies could be conducted with the alkaloid Pipermethysticine and the chalcone Flavokavain B. CYP450 profiling of individuals involved in such studies could clarify pharmacogenomic differences in kava metabolism and could be performed either genotypically or phenotypically using substrates such as debrisoquine for CYP2D6 in humans [54].
